# Supplementary material for: Modeling glioblastoma heterogeneity as a dynamic network of cell states
Source: Mol Syst Biol. 2021 Sep 16;17(9):e10105. doi: 10.15252/msb.202010105 (PMC8444284; doi:10.15252/msb.202010105)
Supplement: Supplementary file 6 — Source Data for Figure 5 [file MSB-17-e10105-s004.zip › Figure5A_sourcedata/GSEA_3071/hallmarks_stateV.GseaPreranked.1623742546329/HALLMARK_REACTIVE_OXYGEN_SPECIES_PATHWAY.html]

Details for gene set HALLMARK\_REACTIVE\_OXYGEN\_SPECIES\_PATHWAY[GSEA]

|  || Dataset | state43071 |
| Phenotype | NoPhenotypeAvailable |
| Upregulated in class | na\_neg |
| GeneSet | HALLMARK\_REACTIVE\_OXYGEN\_SPECIES\_PATHWAY |
| Enrichment Score (ES) | -0.5789705 |
| Normalized Enrichment Score (NES) | -2.2101917 |
| Nominal p-value | 0.0 |
| FDR q-value | 0.0 |
| FWER p-Value | 0.0 |
Table: GSEA Results Summary

  

Fig 1: Enrichment plot: HALLMARK\_REACTIVE\_OXYGEN\_SPECIES\_PATHWAY      
 Profile of the Running ES Score & Positions of GeneSet Members on the Rank Ordered List

  

| PROBE | GENE SYMBOL | GENE\_TITLE | RANK IN GENE LIST | RANK METRIC SCORE | RUNNING ES | CORE ENRICHMENT || 1 | PRDX2 |  |  | 105 | 1.355 | 0.0444 | No |
| 2 | CDKN2D |  |  | 190 | 1.196 | 0.0853 | No |
| 3 | GPX4 |  |  | 701 | 0.746 | 0.0240 | No |
| 4 | SCAF4 |  |  | 2714 | 0.298 | -0.3435 | No |
| 5 | TXNRD2 |  |  | 3583 | -0.312 | -0.4934 | No |
| 6 | ATOX1 |  |  | 3627 | -0.320 | -0.4863 | No |
| 7 | MBP |  |  | 3694 | -0.330 | -0.4832 | No |
| 8 | SRXN1 |  |  | 3924 | -0.371 | -0.5089 | No |
| 9 | LAMTOR5 |  |  | 3941 | -0.376 | -0.4941 | No |
| 10 | JUNB |  |  | 4027 | -0.394 | -0.4915 | No |
| 11 | TXNRD1 |  |  | 4077 | -0.404 | -0.4816 | No |
| 12 | GLRX2 |  |  | 4553 | -0.566 | -0.5448 | No |
| 13 | PFKP |  |  | 4734 | -0.666 | -0.5473 | Yes |
| 14 | PRDX4 |  |  | 4821 | -0.732 | -0.5288 | Yes |
| 15 | G6PD |  |  | 4945 | -0.861 | -0.5113 | Yes |
| 16 | GCLM |  |  | 4957 | -0.874 | -0.4719 | Yes |
| 17 | TXN |  |  | 5054 | -1.035 | -0.4409 | Yes |
| 18 | FTL |  |  | 5072 | -1.077 | -0.3930 | Yes |
| 19 | PRDX1 |  |  | 5104 | -1.163 | -0.3436 | Yes |
| 20 | LSP1 |  |  | 5113 | -1.190 | -0.2886 | Yes |
| 21 | PDLIM1 |  |  | 5114 | -1.191 | -0.2320 | Yes |
| 22 | GLRX |  |  | 5121 | -1.212 | -0.1756 | Yes |
| 23 | NQO1 |  |  | 5210 | -1.596 | -0.1165 | Yes |
| 24 | MGST1 |  |  | 5286 | -2.788 | 0.0017 | Yes |
Table: GSEA details [plain text format]

  

Fig 2: HALLMARK\_REACTIVE\_OXYGEN\_SPECIES\_PATHWAY: Random ES distribution      
 Gene set null distribution of ES for **HALLMARK\_REACTIVE\_OXYGEN\_SPECIES\_PATHWAY**
